# Rhesus Brain Transcriptomic Landscape in an *ex vivo* Model of the Interaction of Live *Borrelia Burgdorferi* With Frontal Cortex Tissue Explants

**DOI:** 10.3389/fnins.2019.00651

**Published:** 2019-06-28

**Authors:** Zhe Ding, Mingbiao Ma, Lvyan Tao, Yun Peng, Yuanyuan Han, Luyun Sun, Xiting Dai, Zhenhua Ji, Ruolan Bai, Miaomiao Jian, Taigui Chen, Lisha Luo, Feng Wang, Yunfeng Bi, Aihua Liu, Fukai Bao

**Affiliations:** ^1^Yunnan Province Key Laboratory for Tropical Infectious Diseases in Universities, Kunming Medical University, Kunming, China; ^2^Department of Microbiology and Immunology, Kunming Medical University, Kunming, China; ^3^Department of Biochemistry and Molecular Biology, Kunming Medical University, Kunming, China; ^4^Institute of Medical Biology, Chinese Academy of Medical Sciences and Peking Union Medical College, Kunming, China; ^5^Yunnan Province Integrative Innovation Center for Public Health, Diseases Prevention and Control, Kunming Medical University, Kunming, China; ^6^Yunnan Demonstration Base of International Science and Technology Cooperation for Tropical Diseases, Kunming, China

**Keywords:** transcriptomic analysis, innate immune system, neuroinflammation, CNS, *Borrelia burgdorferi*, Lyme neuroborreliosis, FOLR2

## Abstract

Lyme neuroborreliosis (LNB) is the most dangerous manifestation of Lyme disease caused by the spirochete *Borrelia burgdorferi* which can reach the central nervous system most commonly presenting with lymphocytic meningitis; however, the molecular basis for neuroborreliosis is still poorly understood. We incubated explants from the frontal cortex of three rhesus brains with medium alone or medium with added live *Borrelia burgdorferi* for 6, 12, and 24 h and isolated RNA from each group was used for RNA sequencing with further bioinformatic analysis. Transcriptomic differences between the *ex vivo* model of live *Borrelia burgdorferi* with rhesus frontal cortex tissue explants and the controls during the progression of the infection were identified. A total of 2249, 1064, and 420 genes were significantly altered, of which 80.7, 52.9, and 19.8% were upregulated and 19.3, 47.1, 80.2% were downregulated at 6, 12, and 24 h, respectively. Gene ontology and KEGG pathway analyses revealed various pathways related to immune and inflammatory responses during the spirochete infection were enriched which is suggested to have a causal role in the pathogenesis of neurological Lyme disease. Moreover, we propose that the overexpressed FOLR2 which was demonstrated by the real-time PCR and western blotting could play a key role in neuroinflammation of the neuroborreliosis based on PPI analysis for the first time. To our knowledge, this is the first study to provide comprehensive information regarding the transcriptomic signatures that occur in the frontal cortex of the brain upon exposure to *Borrelia burgdorferi*, and suggest that FOLR2 is a promising target that is associated with neuroinflammation and may represent a new diagnostic or therapeutic marker in LNB.

## Introduction

Lyme disease is identified as a multistage and multisystem disease which results in the neurological manifestation of systemic infection at a later period, which is called LNB (Pachner and Steiner, 2007). This spirochete, *Borrelia burgdorferi*, with characteristic features such as neural invasion, is the primary pathogen of Lyme disease and can attack the CNS and PNS, leading to lymphocytic meningitis, cranial neuritis, radiculitis, and other focal or multifocal mononeuropathies (Hansen et al., 2013; Beuchat et al., 2018; Halperin, 2018). Generally, patients may have an impairment in cognition and memory (Halperin, 1997). It has been reported that immune-mediated neuronal and glial cell damage, including elevated levels of proinflammatory cytokines and chemokines, may be involved in the neuropathogenesis of LNB (Ramesh et al., 2013a). Due to the increasing prevalence of Lyme disease around the world (Koedel et al., 2015), an improved understanding of the neuropathogenesis resulting from gene interactions in LNB is warranted for the development of better therapeutic strategies to prevent this disease.

Given the similarity of pathology and disease progression of Lyme disease between humans and non-human primates (NHPs), an NHP model of LNB has been widely developed. Rhesus macaques (*Macaca mulatta*) are ideal models for studies related to human infectious diseases, and they are widely used in studies of neurobiology and psychobiology (Xue et al., 2016). In addition, the adaptability of the rhesus monkey and its overall genetic and physiological resemblance to humans (Bimber et al., 2017) makes this species appropriate for studying the immunological response to *B*. *burgdorferi* in the brain. While much progress has been made in investigating the immune response to Lyme disease using animal models, including mouse (Bramwell et al., 2014; Byram et al., 2015; Hansen et al., 2016; Sallay et al., 2017), hamster (Croke et al., 2000; Munson et al., 2012), rabbit (Shang et al., 2000; Scott et al., 2017), and dog (Smith et al., 2012; Wagner et al., 2015), these experimental animals can exhibit erythema migrans or arthritis. However, the rhesus macaque model plays an indispensable role in studies related to neuroborreliosis (Roberts et al., 1998; Pachner et al., 2001), as the overall understanding of the inflammation response leading to LNB remains elusive.

Transcriptomic analysis can provide insights into the molecular basis underlying the development of Lyme disease (Iyer and Schwartz, 2016; Casselli et al., 2017; Marques et al., 2017; Popitsch et al., 2017), and the differentially gene expression profile has implications in the pathogenesis and, hence, potential treatment strategies to combat this disease (Bouquet et al., 2016). Overall, previous studies have helped define the roles of sustained differential gene expression signatures in Lyme disease that may be useful in the future for diagnosis or treatment. We hypothesize that there are some complex interplay among some genes and distinct signaling pathways initiated by spirochetes in the brain which could serve as the underlying molecular and genetic basis of LNB. In this study, we used Illumina Hiseq 2500 to compare the gene expression profiles of frontal cortex brain explants that were exposed to live spirochetes (*B. burgdorferi*), along with the controls, to investigate the regulatory mechanism underlying inflammation in host immune responses at sequential time points in three rhesus macaques.

## Materials and Methods

### Animals

Three rhesus macaques weighing 1.5 to 3 kg (*Macaca mulatta*) of Chinese origin Monkey 1 (Male; age, 1 year old), Monkey 2 (Male; age, 1 year old), and Monkey 3 (Female; age, 1 year old) were from the Institute of Medical Biology, Chinese Academy of Medical Sciences and Peking Union Medical College. Animal permit number: SCXK (DIAN) K2015-0004. Frontal cortex tissues for the *ex vivo* experiments were given as a present for our laboratory and were collected from these rhesus macaques that were slated for euthanasia. These animals were healthy and were all uninfected with *B. burgdorferi*. All efforts were made to minimize suffering, and all procedures were performed in accordance with the rules for euthanasia of experimental animals in the Chinese experimental animal information network. This experiment was performed in accordance with the Guide for the Care and Use of Laboratory Animals (Carbone, 2012) and the ARRIVE Guidelines for Reporting Animal Research (Kilkenny et al., 2010). The Animal Ethical and Welfare Committee of Kunming Medical University reviewed and approved this study, and the necropsy was conducted by qualified experimenters.

### Growth and Preparation of Live Spirochetes

The low-passage-number *B. burgdorferi* strain 4680 (*Bb*4680, DSMZ, A1511358-1) was grown in Barbour-Stoenner-Kelly II (BSK II) medium (Ruzic-Sabljic et al., 2006; Zuckert, 2007) supplemented with 6% rabbit serum (Thermo Fisher Scientific, United States) to the late logarithmic phase under microaerophilic conditions. The cultures were incubated at 37°C and examined by dark-field microscopy for the plate count. After centrifugation (4°C, 10 min, 2000 ×*g*), the supernatant was discarded. After the cells were washed twice with sterile PBS, an appropriate volume of 10% FBS-RPMI 1640 medium (Thermo Fisher Scientific, United States) was added to a final bacterial concentration of 1 × 10^7^ bacteria/mL.

### Frontal Cortex Brain (FCB) Explant Co-culture With Live Spirochetes and Experimental Design

Freshly harvested frontal cortex tissues were collected immediately at necropsy from three rhesus macaques and washed twice with PBS. The frontal cortex was sliced as thin as possible using sterile scalpel handle (J11060, Jinzhong^®^, Shanghai), scalpel blades (J0B080, Jinzhong^®^, Shanghai) and atraumatic tissue forceps (JYF010, Jinzhong^®^, Shanghai). Each section weighed 0.5 g and placed in a separate T-25 flask (Corning, NY, United States) containing 4 mL of 10% FBS-RPMI 1640 medium or 4 mL *B*. *burgdorferi* (*Bb*) bacteria suspension prepared as previously described. We ensured that each tissue section was totally soaked in the medium and triplicate tissue sections from three animals were then incubated in a humidified incubator with 5% CO2 at 37°C for 6, 12, and 24 h, respectively. After 6, 12, and 24 h of incubation, the co-culture FCB sections of the three rhesus macaques were collected and stored at -80°C.

### RNA Isolation

Equivalent amounts of brain tissue weighed 20 mg were gently removed and lysed with 1 ml of TRIZOL Reagent (Invitrogen) in the triturator. RNA was then extracted with chloroform–isopropanol. After 30 min of incubation, the samples were centrifuged to separate the RNA, which was subsequently washed with 75% ethanol. The RNA concentration and purity was measured using a NanoDrop-2000 spectrophotometer (Thermo Scientific^TM^), and the qualified samples (1.7 < OD260/OD280 < 2.0) were stored at -80°C. Possible DNA contamination was removed by subjecting the RNA to DNase treatment (5 × g DNA Eraser Buffer, Takara).

### Reverse Transcription

RNA was digested with 5 × gDNA Eraser Buffer (Takara) for 5 min at room temperature and then reverse transcribed into cDNA using the PrimeScript^TM^ RT reagent Kit (Takara). The reaction mixture contained the following: 1 μg of RNA, 4 μL of 5 × PrimeScript Buffer 2 (for real-time PCR), 1 μL of PrimeScript RT Enzyme Mix I, and RNase Free dH2O to a final volume of 20 μL. The reaction was performed in a Bio-Rad C1000 Touch^TM^ Thermal Cycler under the following conditions: 37°C for 15 min, 85°C for 5 s, 4°C infinitely. The cDNA samples were stored at -20°C.

### cDNA Library Preparation and Sequencing

The cDNA library was established using the RNA-Seq Library Preparation Kit (Illumina) following the manufacturer’s protocol. In brief, poly-(A) mRNA was separated from 5 μg of total RNA using the Oligo d(T) magnetic beads. Then, after the fragmentation buffer was added, the mRNA was fragmented into small pieces under an elevated temperature. Using the mRNA fragments as templates, the first-strand cDNA was synthesized with 6-base random primers. Next, the second-strand cDNA was synthesized using DNA polymerase I and RNase H. The obtained cDNAs were end-repaired by polymerase and ligated with “A-tailing” base adaptors. Target fragments were selected by magnetic beads for PCR amplification to construct the final cDNA library and the final double-stranded cDNA samples were examined by agarose electrophoresis. After the library was quantified using Qubit 2.0 (Illumina), sequencing was performed on an Illumina HiSeq 2500 sequencing platform.

### RNA-Seq Data Processing

To identify the genes that are involved in the response to the inflammation response in the brain, we performed a time course of *Bb* infection in the FCB tissue of rhesus monkeys, and the RNA was isolated for analysis using the Illumina HiSeq 2500 sequencing technique at 6, 12, and 24 h after the co-culture experiment, as previously described. The original image data obtained from the Illumina Hiseq sequencing was converted into sequence data through Base Calling in the FASTQ format, and the raw data were obtained. The quality control of the raw data were performed using FastQC, and the raw data were pre-processed using Trimmomatic (Illumina) software to remove the adaptor sequences, ribosomal RNA, and other contaminants that could potentially interfere with the clustering and assembly. The resulting clean data were compared with the corresponding reference genome to obtain comprehensive transcriptome information, and the RPKM (Wagner et al., 2012) in RNA-seq data was obtained for analyzing DEGs. An RPKM ≥ 0.5 was considered statistically significant.

### Bioinformatics Analysis

A heatmap was generated that illustrated the significant alterations in gene expression between the *Bb* co-culture with FCB explants and the controls at 6, 12, and 24 h. We defined significantly altered genes as those genes with a fold change (FC) ≥ 2 and FDR (False Discovery Rate) < 0.01, which was the correction of the *P*-value using the Benjamini–Hochberg method (Madar and Batista, 2016). Functional classifications of the DEGs were determined with GO annotation using the online DAVID tool, and the pathway analysis of the DEGs was performed with the KEGG database. Based upon the functional annotation in the GO database, the DEGs were assigned to categories of various BP, CC, and MF. Significant enrichment was defined as a *P*-value < 0.05. The results of the analysis of enrichment of GO and KEGG are also presented in this study. In addition, a hierarchical cluster analysis was performed for the enriched genes by Cluster software, and the PPI network of the proteins encoded by the DEGs was searched using STRING online software. A network layout was produced by Cytoscape software, using the BiNGO 3.03 Cytoscape plug-in to assess overrepresentation of GO categories in biological networks (Maere et al., 2005).

### Confirmation of Expression by Real-Time PCR

Real-time PCR was performed using Takara SYBR^®^ Premix Ex Taq II (Tli RNaseH Plus) (2×) on a CFX96 Real-Time PCR Detection System. The reaction volume was 25 μL, containing 12.5 μL of Takara SYBR^®^ Premix Ex Taq II (Tli RNaseH Plus) (2×), 1 μL of each primer and 2 μL of the cDNA template. Amplification conditions were as follows: 95°C for 30 s, followed by 40 cycles of 95°C for 5 s, 64°C (55°C for GAPDH) for 30 s. SYBR^®^ Green PCR mix and primers that were specific to our target gene were used to efficiently amplify the target region. The primers used were as follows: GAPDH sense: 5′-GCACCACCAACTGCTTAGCAC-3′; GAPDH antisense: 5′-TCTTCTGGGTGGCAGTGATG-3′; FOLR2 sense: 5′-ACTTCTGCTGCTTCTGGTCT-3′; FOLR2 antisense: 5′-GTCATGCAGCTTGTCCTCAG-3′.

### Confirmation of Expression by Western Blotting

The total protein from each brain tissue sample was extracted using RIPA lysis buffer (Solarbio, #R0010) supplemented with the protease inhibitor PMSF (Solarbio, #R0010). After the protein concentration was determined using the Bicinchoninic acid (BCA) assay (Reichelt et al., 2016), the protein and 4 × Protein SDS PAGE Loading Buffer (Takara, #9173) were mixed in a ratio of 3:1 in boiling water for 15 min, and then the samples were stored at -20°C. Next, 60 μg protein samples were separated using 10% TGX stain-free^TM^ FastCast^TM^ Acrylamide gel (Takara) (Gilda and Gomes, 2013; Posch et al., 2013; Ghosh et al., 2014; Gilda and Gomes, 2015) electrophoresis and transferred to a polyvinyl difluoride membrane. After being blocked with 5% non-fat dry milk, the membranes were incubated overnight at 4°C with a FOLR2 primary antibody (LifeSpan, LS-C152098-50). Then, the samples were incubated with a goat anti-rabbit horseradish peroxidase-labeled secondary antibody (Abcam, ab6721) for 2 h at room temperature. The resulting immunocomplexes were visualized with chemiluminescence using the Clarity Western ECL Substrate (Bio-Rad), and the signal intensity was quantified with the Image Lab^TM^ (Bio-Rad) software version 5.2.1. Densitometry was normalized to the corresponding total protein of the control.

### Statistical Analysis

The data are presented as the means ± standard error (SEM). All calculations were performed using the software package GraphPad PRISM version 6.01 (GraphPad Software, Inc.). All experiments were performed in triplicate unless stated otherwise. An unpaired two-tail Student’s *t*-test was performed, as appropriate, to evaluate the *P*-values, and a *P*-value < 0.05 was considered statistically significant.

## Results

### Quality Control and Correlations of the Data

**FIGURE 1:**
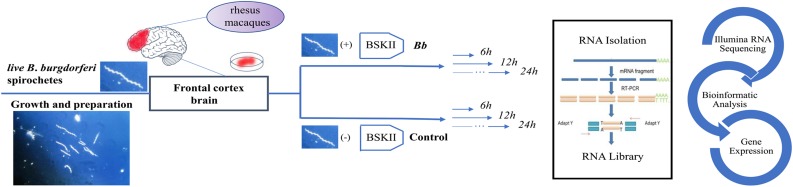
Schematic description of the experimental design. The asterisk represents the *Borrelia burgdorferi* spirochetes.

**FIGURE 2:**
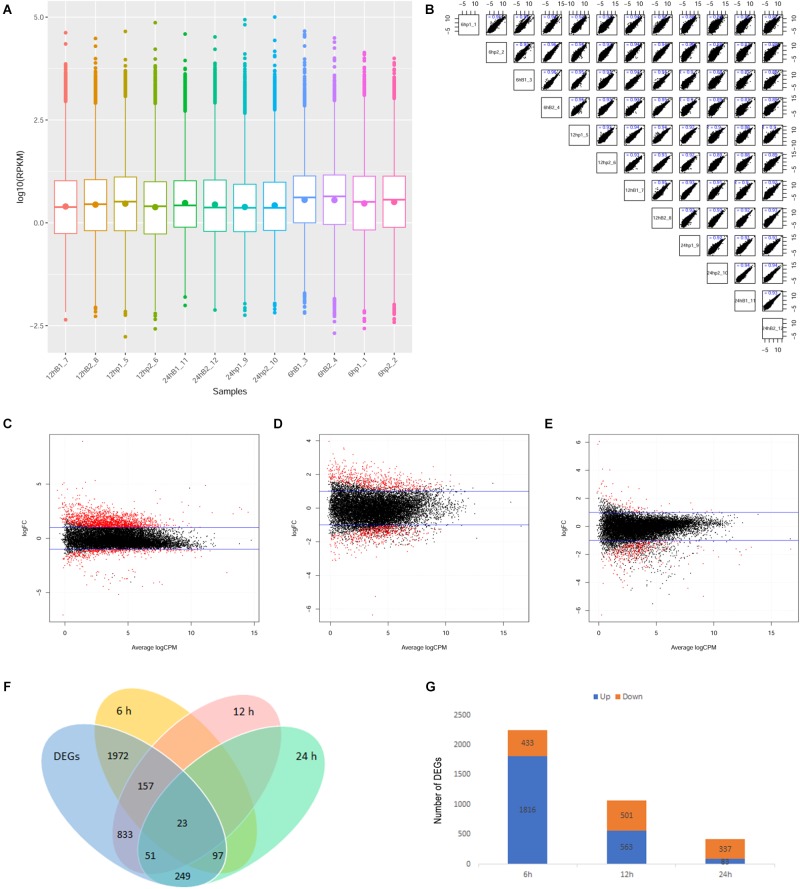
Overview of mRNA profiling in this study. **(A)** Boxplot of the gene expression levels of various samples. Box charts for each region correspond to the maximum, upper quartile, median, lower quartile, and minimum of log10 (RPKM). **(B)** Scatter plot of the gene expression correlation in this study. The abscissa and ordinate represent log10(RPKM) of different sample genes respectively. **(C–E)** MA plot indicating upregulated and downregulated differentially expressed genes (DEGs) in the *ex vivo* model 6, 12, and 24 h after *Bb* infection. The abscissa represented Average log2 (CPM). The ordinate represented log2 (FC). The two blue lines represent the threshold of differentially expressed genes and the points outside the threshold are marked in red. log2 (FC) > 1 represents upregulated and log2 (FC) < -1 represents downregulated. **(F)** Venn diagram representing the overlap of identified DEGs. Overlaps showing 6 h-DEGs (*n* = 2249; yellow), 12 h-DEGs (*n* = 1064; red), 24 h-DEGs (*n* = 420; green), 6, 12, 24 h-DEGs (*n* = 3382; blue). **(G)** Number of upregulated and downregulated DEGs at 6, 12, and 24 h.

The experimental process is shown in Figure 1. The gene expression levels in the rhesus macaque frontal cortex after co-culture with *Bb* or medium alone at the indicated time points are shown in Figure 2A. The ratio of clean reads as a percentage of all raw reads was greater than 93% for all samples. This suggests that the data were high-quality and suitable for subsequent analysis. Additionally, the correlation of the gene expression level between samples is shown in Figure 2B, which is an important index to test the reliability of the experiment and the reasonableness of the sample selection. In our study, the *R*^2^ (square of Pearson correlation coefficient) of all groups was greater than 0.8, thus the reliability of subsequent experiments was guaranteed.

### DEGs in the Frontal Cortex Brain Transcriptome Following *Bb* Infection

**FIGURE 3:**
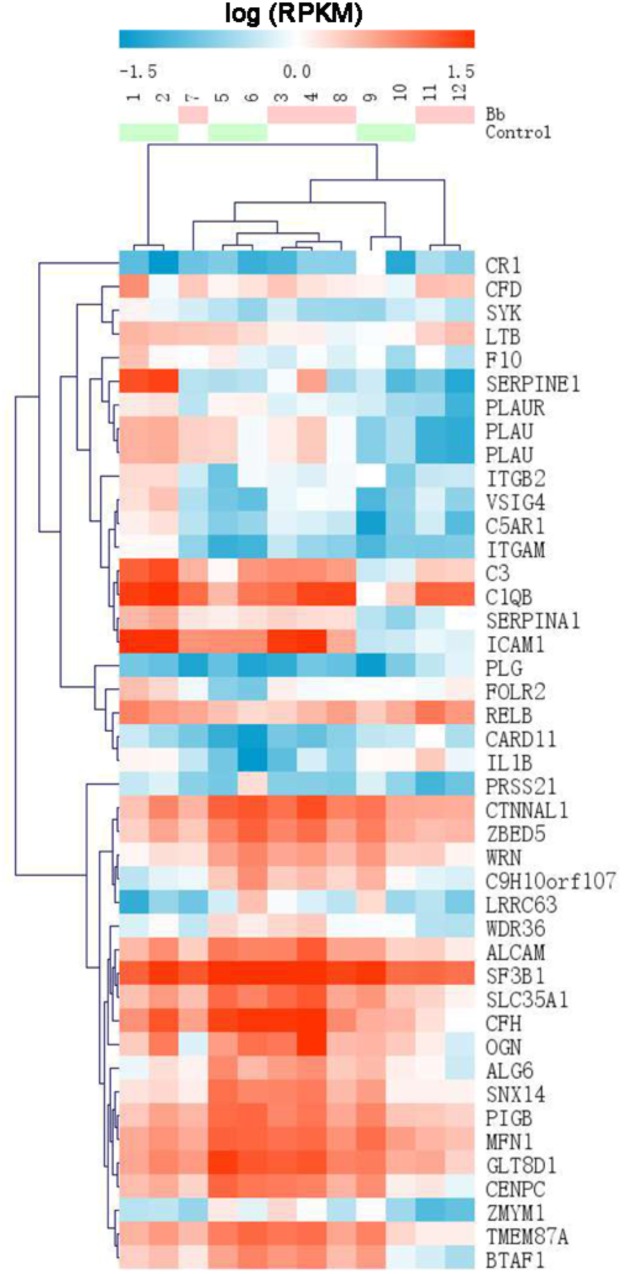
Heatmap of DEGs identified by RNA sequencing showing the hierarchical clustering of the relative expression of a portion of the DEGs in each sample, for the sake of brevity. The *Bb* group and control group are represented by red and green, respectively. High and low abundance of gene expression are shown in red and blue, respectively.

Using an MA plot, we could clearly identify DEGs at separate time points (Figure 2C–E). The details of these DEGs at each time point are listed in the respective tables (Additional files 1–3: Supplementary Tables S1–S3). The Venn diagram showed that the number of DEGs at 6, 12, and 24 h was 2,249, 1,064, and 420, respectively (Figure 2F). A comparison of DEGs at each time point revealed that 23 DEGs were shared at all three time points (Figure 2F), and 1,816 (80.7%) genes were identified as being significantly upregulated and 433 (19.3%) were significantly downregulated at 6 h after the *Bb* infection, 563 genes (52.9%) were significantly upregulated and 501 genes (47.1%) were significantly downregulated at 12 h after the *Bb* infection, while 83 genes (19.8%) were significantly upregulated and 337 genes (80.2%) were significantly downregulated at 24 h after the *Bb* infection (Figure 2G). Moreover, the heatmap (Figure 3) illustration of the DEGs shows the changes in mRNA abundance observed in each group following the *Bb* infection.

### GO Functional Analysis of DEGs

**FIGURE 4:**
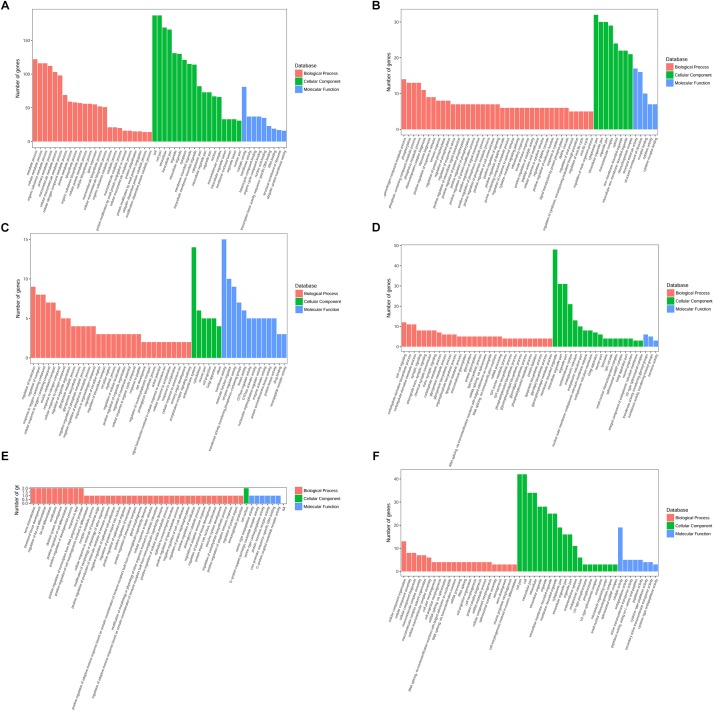
GO annotation analysis of upregulated and downregulated DEGs at various time points. GO annotation characterization of the molecular functions, biological processes, and cellular components based on significantly upregulated or downregulated DEGs. **(A)** GO annotation for upregulated DEGs at 6 h. **(B)** GO annotation for downregulated DEGs at 6 h. **(C)** GO annotation for upregulated DEGs at 12 h. **(D)** GO annotation for downregulated DEGs at 12 h. **(E)** GO annotation for upregulated DEGs at 24 h. **(F)** GO annotation for downregulated DEGs at 24 h.

The upregulated DEGs at 6 h were enriched in BP terms including protein ubiquitination, lipoprotein metabolic process and protein modification by small protein conjugation, CC terms including cytoplasm, and MF terms including ubiquitin-protein transferase activity and RNA binding. The upregulated DEGs at 12 h were enriched in BP terms including the glutamate receptor signaling pathway and G-protein coupled acetylcholine receptor signaling pathway, CC terms including neuron component, and MF terms such as protein kinase activity. The upregulated DEGs at 24 h were enriched in BP terms including regulation of the adaptive immune response, CC terms including cell surface, and MF terms including protein-hormone receptor activity. The GO enrichment terms of BP, CC, and MF for upregulated DEGs at 6, 12, and 24 h are shown in Figure 4A,C,E.

Meanwhile, the downregulated DEGs at 6 h were enriched in BP terms including alpha-beta T cell activation, cellular response to IFN-γ, cellular response to IL-1, and positive regulation of leukocyte differentiation, CC terms including cytosolic ribosome, and MF terms including CCR chemokine receptor binding and chemokine activity. The downregulated DEGs at 12 h were enriched in BP terms including GPI anchor metabolic process and synaptic signaling, CC terms including endoplasmic reticulum part, and MF terms such as neuropeptide hormone activity. The downregulated DEGs at 24 h were enriched in BP terms including semaphorin-plexin signaling pathway, CC terms including prespliceosome, and MF terms including active transmembrane transporter activity. The GO enrichment terms of BP, CC, and MF for downregulated DEGs at 6, 12, and 24 h are shown in Figure 4B,D,F.

The complete GO results for upregulated and downregulated genes 6, 12, and 24 h after *Bb* infection are shown in the Additional files 4–9: Supplementary Table S4–S9.

### KEGG Pathway Enrichment Analysis of DEGs

**FIGURE 5:**
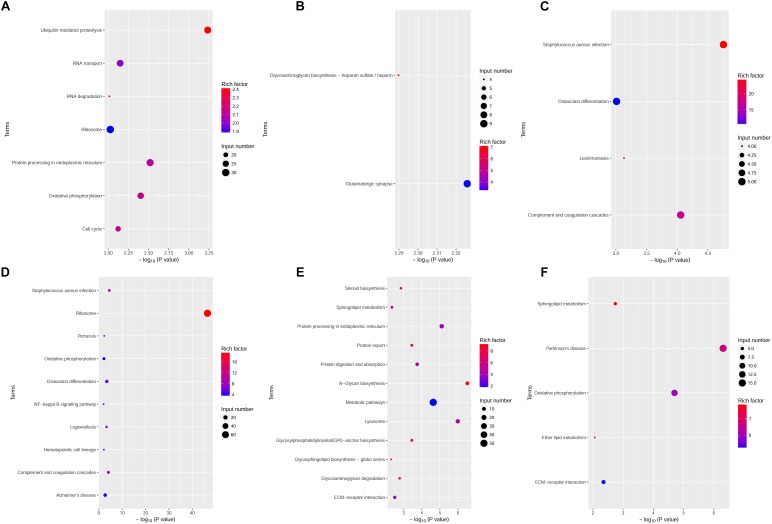
KEGG pathway enrichment analysis of upregulated and downregulated DEGs at various time points. The dot size and depth indicate the number of DEGs contained in the KEGG pathway and the degree of enrichment of the rich factor, respectively. We chose the most significant top 20 GO terms according to –log10 (*P*-value). **(A–C)** KEGG analysis of upregulated DEGs at 6, 12, and 24 h. **(D–F)** KEGG analysis of downregulated DEGs at 6, 12, and 24 h. Rich factor: the enrichment factor of KEGG pathway enrichment analysis of DEGs; Input number: the number of genes enriched in this pathway in the list of differentially expressed genes.

The DEGs between the *Bb* group and the control group at each time point were subjected to KEGG pathway enrichment analysis through the KEGG database. The threshold for significant enrichment was set at a *P*-value < 0.01. According to the KEGG pathway enrichment analysis, the upregulated DEGs at 6 h were significantly enriched in 7 signaling pathways, including protein processing in the endoplasmic reticulum (Figure 5A). The upregulated DEGs at 12 h were significantly enriched in 2 signaling pathways including glutamatergic synapse and glycosaminoglycan biosynthesis – heparan sulfate/heparin (Figure 5B). Moreover, the upregulated DEGs at 24 h were significantly enriched in 4 signaling pathways, including *Staphylococcus aureus* infection and complement and coagulation cascades (Figure 5C). The downregulated DEGs at 6 h were significantly enriched in 10 signaling pathways, including complement and coagulation cascades, the NF-kappa B signaling pathway and AD (Figure 5D). The downregulated DEGs at 12 and 24 h were significantly enriched in 12 signaling pathways and 5 signaling pathways, respectively (Figure 5E,F). Interestingly, sphingolipid metabolism and ECM-receptor interactions were both enriched in the downregulated DEGs at 12 and 24 h. Complement and coagulation cascades, which were primarily related to immune and inflammatory responses, were enriched in the DEGs at 6 and 24 h.

### Prediction of FOLR2 Closely Related to Different Branches of the Immune System Based on PPI Network Analysis

**FIGURE 6:**
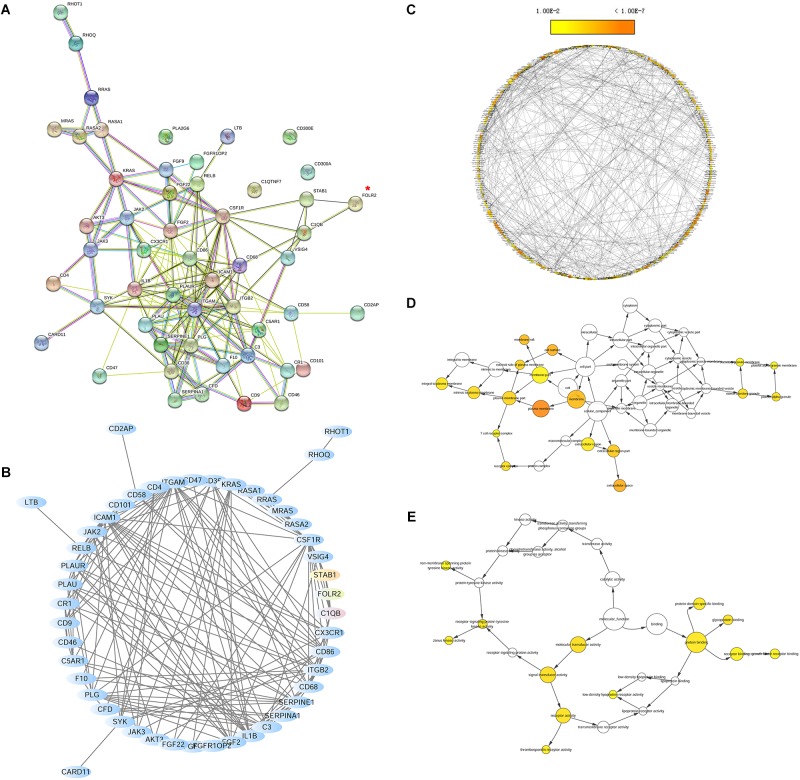
The network analysis of the biological interactions within DEGs based on the RNA-seq datasets. **(A)** The PPI network of DEGs related to immune responses. The network nodes represent proteins and the edges represent protein-protein associations. The position of FOLR2 is marked by a red asterisk. **(B)** The network of DEGs related to immune responses using Cytoscape. The first neighbors of FOLR2 (red) were STAB1 (yellow) and C1QB (green). **(C)** Representative biological process based on BiNGO. **(D)** Representative cellular component based on BiNGO. **(E)** Representative molecular function based on BiNGO. Orange categories are most significantly overrepresented and white nodes are not significantly overrepresented. The area of a node was proportional to the number of DEGs in the test set.

To investigate the interaction of DEGs in response to *Bb* infection following the co-culture experiment, the PPI network analysis was performed using STRING. As shown, the predicted PPI in the frontal cortex brain of the rhesus macaques exhibited an intricate network after the *Bb* infection (Figure 6A). The established PPI network (PPI enrichment *p*-value < 1.0e-16) contained 52 nodes and 168 edges. In addition, 48 pathways were significantly enriched using the FDR value < 0.01 as the threshold of significant enrichment, including the Complement and coagulation cascades, Ras signaling pathway, MAPK signaling pathway, Rap1 signaling pathway, and NF-kappa B signaling pathway, which were closely interrelated as components of the immune response in the progression of the *Bb* infection (Table 1 and Additional file 10: Supplementary Table S10). Moreover, FOLR2 was shown to be located at an important position from which different branches of the immune response could be driven in the PPI network. To investigate the molecular interaction networks, the predominant function of the DEGs was analyzed using BiNGO 3.03 Cytoscape plug-in, and the intuitive and customizable visual representation of the results was showed in Figure 6B–E. The results of ontological analysis are shown in Table 2. It shows that the first neighbors of FOLR2 were STAB1 and C1QB and the interactions between the DEGs were multitudinous. According to the PPI analysis, the co-expression of FOLR2-STAB1 (score: 0.413) and FOLR2-C1QB (score: 0.329) could potentially activate a series of reactions; however, further studies are warranted.

**Table 1:** KEGG pathways analysis in the PPI network.

Pathway ID	Pathway description	Count in gene set	False discovery rate (FDR)	–LOG10(FDR)
4610	Complement and coagulation cascades	12	3.49E-17	16.45717457
5150	*Staphylococcus aureus* infection	8	8.34E-11	10.07883395
4014	Ras signaling pathway	10	1.65E-08	7.78251606
4010	MAPK signaling pathway	10	4.67E-08	7.33068312
4015	Rap1 signaling pathway	9	9.93E-08	7.00305075
4640	Hematopoietic cell lineage	7	9.93E-08	7.00305075
4064	NF-kappa B signaling pathway	7	1.00E-07	7.00000000
5152	Tuberculosis	8	3.81E-07	6.41907502
5140	Leishmaniasis	6	6.78E-07	6.16877031
5205	Proteoglycans in cancer	8	2.11E-06	5.67571754


*The top 10 pathways significantly enriched in the PPI network sorted by false discovery rate (FDR, Benjamini–Hochberg correction). The *p*-value < 0.01 was set as the threshold of significant enrichment. The whole results were shown in Additional file 10: Supplementary Table S10.*

**Table 2:** Ontological analysis.

GO-ID	Description	*p*-value	Correction *p*-value
**Biological process**			
9611	Response to wounding	2.25E-17	3.01E-14
2684	Positive regulation of immune system process	8.84E-15	5.92E-12
23052	Signaling	3.51E-12	1.57E-09
2682	Regulation of immune system process	8.19E-12	2.74E-09
48583	Regulation of response to stimulus	1.31E-11	3.50E-09
**Cellular component**			
5886	Plasma membrane	6.42E-11	8.42E-09
5615	Extracellular space	5.28E-08	3.46E-06
9986	Cell surface	9.53E-08	4.16E-06
16020	Membrane	3.71E-07	1.07E-05
45121	Membrane raft	4.07E-07	1.07E-05
**Molecular function**			
60089	Molecular transducer activity	2.86E-06	3.06E-04
4871	Signal transducer activity	2.86E-06	3.06E-04
1948	Glycoprotein binding	8.32E-06	4.67E-04
4872	Receptor activity	8.74E-06	4.67E-04
4716	Receptor signaling protein tyrosine kinase activity	1.33E-05	5.70E-04


*The top 5 categories significantly overrepresented using BiNGO plugin according to the adjusted *p*-value. The *p*-value < 0.01 was set as the threshold of significant enrichment.*

### Validation of mRNA and Protein Expression of FOLR2 Using qPCR and Western Blotting

**FIGURE 7:**
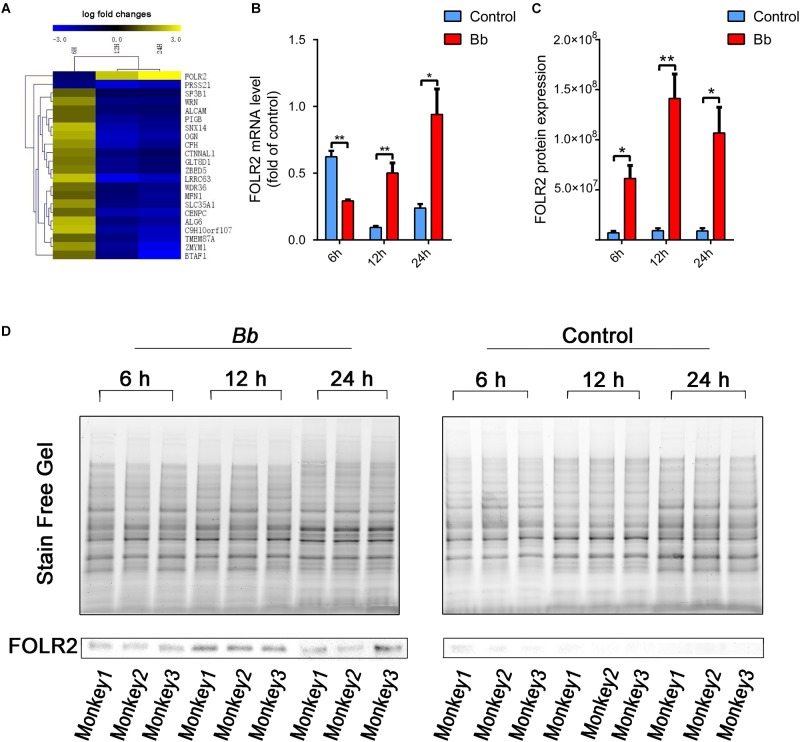
Significant alteration of FOLR2 expression during the progressions of *Bb* infection. **(A)** Heatmap showing the fold changes of DEGs based on RNA-seq datasets at three time points in the frontal cortex co-cultured with live *Bb* compared with the control. Upregulated and downregulated genes are shown in yellow and blue, respectively. **(B)** Real-time PCR analysis of FOLR2 expression comparing explants from the frontal cortex of rhesus brains co-cultured with live *Bb* with the controls at 6, 12, and 24 h. **(C)** Protein expression of FOLR2 in explants from the frontal cortex of rhesus brains co-cultured with live *Bb* and the controls at 6, 12, and 24 h, validated by western blotting. **(D)** Western blotting results using total protein loading in the stain-free gel as a loading control. ^∗^*p* < 0.05, ^∗∗^*p* < 0.01, *Bb* vs. Control, analyzed with an unpaired two-tail Student *t*-test. Data were expressed as the means ± SEM.

Throughout the whole infection course in our study, the expression changes of FOLR2 were the most significant among all the DEGs which inevitably attracted our attention (Figure 7A). Strikingly, the fold change (FC) of FOLR2 mRNA levels at 6, 12, and 24 h according to RNA sequencing were -1.2713, 2.3536, and 5.8588, respectively. Then, real-time PCR was performed to confirm the mRNA expression levels at 6, 12, and 24 h after the *Bb* infection, and the results were consistent with those obtained with RNA-seq (Figure 7B). The mRNA expression level of FOLR2 in the *Bb* group was downregulated compared to the control at 6 h only. In contrast, the mRNA expression level of FOLR2 in the *Bb* group was increasingly upregulated compared to the control at 12 and 24 h. Consistent with the increased level of mRNA expression at 12 and 24 h, the protein level of FOLR2 was also significantly upregulated in cases with *Bb* infection (Figure 7C,D). However, the protein expression of FOLR2 was upregulated at 6 h after *Bb* infection, which was inconsistent with the mRNA level, possibly due to the regulation of translation (Schwanhausser et al., 2013).

## Discussion

*Borrelia burgdorferi*, which is a fairly complicated pathogen, can infect a variety of animals, resulting in systemic infection, including neurological infection, which is perhaps regarded as the most dangerous manifestation of this disease. In the case of persistent *Bb* infection, i.e., if the spirochetes are not cleared out by the host, the nervous system gets attacked, and the inflammation which is elicited by the relentless effort of the host to clear the pathogen is acknowledged as the primary pathogenic mechanism (Ramesh et al., 2017). In the present study, we focused on the alterations of transcriptional profiles to explore the interaction between the host and organism in the CNS via the co-cultivation of live *Bb* and the frontal cortex of three rhesus macaques.

We have shown that distinct changes in the frontal cortex brain transcriptome between control and *Bb* infected tissue samples are observed. Previously, extensive studies of the immune responses to *Bb* during persistent dissemination in the NHP model have helped us to elucidate the neurological deficits (Pachner et al., 2001; Holub et al., 2002). The role of glia cells in initiating or promoting inflammatory responses during *Bb*-induced infection by facilitating the recruitment of peripheral immune cells and producing cytokines or chemokines has been well documented (Ramesh et al., 2008, 2009, 2013b, 2015; Martinez et al., 2015). Concomitant with the activation of microglia, astrocyte, and oligodendrocyte, as well as the production of antibodies, is the cytokines storm that results in neuroinflammation and the neurological sequelae associated with the apoptosis of glial cells and neurons. Consistent with the literatures, our results show that the DEGs related to immune response are significantly upregulated following *Bb* infection such as IL6ST, CSF1R, C1QTNF7, CX3CR1, and CCL24 (Additional files 1–3: Supplementary Table S1–S3) and the complement and coagulation cascades which can regulate the immune response and inflammation are still active 24 h after *Bb* infection comparing control and infected tissue samples (Figure 5C).

Moreover, several studies have provided evidence that chronic spirochetal infection may cause dementia and may be involved in the pathogenesis of neurodegenerative diseases, such as AD (Miklossy et al., 2004; MacDonald, 2007; Miklossy, 2011, 2015). These findings corroborate previous observations that DEGs were significantly enriched in AD, PD, glutamatergic synapse, and sphingolipid metabolism (Figure 5), suggesting that cognitive dysfunction may potentially be a consequence of the impairment of glial and neurons as a result of the interaction between *Bb* and these cells.

The method of co-culture *ex vivo* is commonly used in the NHP model of Lyme disease (Ramesh et al., 2013b, 2017; Martinez et al., 2015) and the results have increased our understanding of the mechanisms during the early and late disseminated infection of *Bb* in the brain. Although the murine model has been recently utilized to trace the spirochetes and neuroinflammation in the brain (Brissette et al., 2012; Divan et al., 2018), the differences between rodents and NHPs are non-negligible; for example, in rodents, *Bb* was colonized in the dura mater rather than pia mater (Divan et al., 2018), and the activation of astrocytes in the rat brain couldn’t be detected (Brissette et al., 2012). In addition, it is questionable whether the various *Bb* strains can infect the brains of mice, considering that the culture results that were obtained in strain 297 and could not be replicated with strain B31 (Divan et al., 2018); this discrepancy could perhaps be illuminated within the perspective of the correlation between strains and disease severity (Wang et al., 2002; Li et al., 2006). For example, the most frequent manifestation of LNB in the US is meningitis, caused by *B*. burgdorferi, sensu stricto rather than *B. garinii* which is characterized by robust inflammatory responses of neuroborreliosis in Europe, most likely due to the differences in antigenic expression (Fallon et al., 2010). Overall, rhesus macaques which can manifest all of the characteristics are the best model of Lyme disease (Cadavid et al., 2003, 2004) including LNB (Bai et al., 2004).

In this study, a high number of significantly perturbed transcripts of FOLR2 was revealed in our transcriptome analysis of live spirochete-stimulated brain tissues. Indeed, FOLR2 was the most significantly altered gene among all DEGs (5.8588-fold increase), and the differential mRNA expression was observed in our real-time PCR results (Figure 7). It is possible that there was an increased translation of exiting transcripts of FOLR2 or the regulation of translation (Schwanhausser et al., 2013), resulting in the persistent overexpression of the protein even at 6 h post-*Bb* infection.

Intriguingly, it is believed that FOLR2 (also referred to as FR-β) is commonly overexpressed in activated macrophages involved in the pathogenesis of inflammation and autoimmune diseases, such as autoimmune encephalomyelitis (Lu et al., 2014), ulcerative colitis (Poh et al., 2017b), atherosclerotic (Muller et al., 2014; Poh et al., 2017b), acute myeloid leukemia (Lynn et al., 2015), lung inflammation (Han et al., 2015), and rheumatoid arthritis (Nogueira et al., 2016; Poh et al., 2017a). In addition, FOLR2 has not been detected in the peripheral blood monocytes in mice, whereas it was observed at sites of inflammation in the macrophages of mice (Shen et al., 2014). Based upon these studies, FOLR2 is a specific marker of the macrophages, and the activated macrophages play a vital role in the development and maintenance of inflammatory diseases. Indeed, functional FOLR2 is predominantly found in CD14^high^CD16^-^ monocytes, which are regarded as the classical subpopulation of proinflammation monocytes (Shen et al., 2014). Based on this premise, folate-targeted imaging and therapeutic agents have been widely introduced into clinical studies as a popular strategy, acting as a locator or targeting drugs selectively to the inflammatory cells (Lu et al., 2015; Poh et al., 2017a,b, 2018).

Transcriptome results obtained from the interaction of *Bb* with the CNS indicate that FOLR2 could be involved in the pathogenesis of neurological Lyme disease, however, to our knowledge FOLR2 is not yet reported in *Bb-* induced infection. Based on the PPI analysis, we hypothesize that the over-expression of FOLR2 may contribute to mediating inflammatory and apoptotic signaling cascades in the CNS following *Bb* infection through the involvement of STAB1 and C1QB in various immune signaling pathways, including the Ras, MAPK, Rap1, PI3K-Akt, NF-kappa B signaling pathway and chemokine signaling pathways (Table 1 and Additional file 10: Supplementary Table S10). MAPK and NF-kappa B signaling have been widely reported to regulate the neuroinflammation and apoptosis in *Bb*-induced CNS infection, contributing to the progression of LNB (Parthasarathy and Philipp, 2014; Martinez et al., 2015). Additionally, C1Q is the initial recognition subcomponent of the classical complement cascade which contributes to the pathology of neuroinflammatory and neurodegenerative in CNS and is expressed by microglia and macrophage (Depboylu et al., 2005). As previously mentioned, FOLR2 is also specifically overexpressed in macrophage based on the previous literature. Our KEGG pathway enrichment analysis indicates that the up-regulated DEGs including C3, PLG, CFD, CR1, and C1QB are still enriched in complement and coagulation cascades, up to 24 h after infection (Figure 5C). It is possible that FOLR2 could play a critical role in initiating and promoting inflammatory and apoptotic signaling cascades, perhaps augmenting the effects of innate immune response against the infectious pathogens through C1QB (the first neighbor) which result in the pathogenesis of CNS Lyme disease.

FOLR2 is a glycosylphosphatidylinositol – anchored membrane protein that mediates the transport of folate which is indispensable in many metabolic pathways, including amino acid interconversions, nucleotide (DNA) synthesis, and methylation reactions, by transporting one-carbon groups (Han et al., 2015). From the pertinent literature, folates play a crucial role in the regeneration and repair of the adult CNS after injury (Iskandar et al., 2010). However, the underlying mechanism of FOLR2 regulation remained elusive.

Here, we present the transcriptomic response of neurons and glial cells to the exposure of *Bb* and the aggregate results from this study reveal that the expression levels of genes involved in immune and defense responses are affected significantly after *Bb* infection. In addition, we propose that FOLR2 is closely related to pathogenesis of *Bb*-induced infection in brain for the first time. The molecular mechanisms of FOLR2 leading to neuroimmune modulation in LNB are lacked and are currently being investigated in our laboratory. Further detailed studies on a cellular level are warranted to gain a better understanding of FOLR2 as well as the crosstalk in a complex immune network that contribute to the pathogenesis of LNB.

## Data Availability

The datasets generated for this study are available on request to the corresponding author.

## Ethics Statement

The study was reviewed and approved by the Animal Ethical and Welfare Committee of KMU. Approval for the animal experimentation was granted by the Guide for the Care and Use of Laboratory Animals and the ARRIVE Guidelines for Reporting Animal Research.

## Author Contributions

ZD conducted all the experiments, analyzed the data, and drafted the manuscript. MM, LT, and YP cultured the spirochetes and established the *ex vivo* model. LS and FB participated in the real-time PCR experiments. FB conceived the project and revised the manuscript. XD and RB coordinated the whole experiments. AL supervised the experiments. YH, ZJ, MJ, TC, LS, and FW provided technical and administrative support. All authors read and approved the final manuscript.

## Conflict of Interest Statement

The authors declare that the research was conducted in the absence of any commercial or financial relationships that could be construed as a potential conflict of interest.

## Abbreviations

AD: Alzheimer’s disease
Bb: Borrelia burgdorferi
BiNGO: the Biological Networks Gene Ontology tool
BP: biological processes
CC: cellular components
CNS: central nervous system
CPM: counts per million reads
DEGs: differentially expressed genes
FBS: fetal bovine serum
FC: fold change
FCB: frontal cortex brain
FDR: False Discovery Rate
GO: Gene Ontology
IFN-γ: interferon-gamma
IL-1: interleukin-1
KEGG: Kyoto Encyclopedia of Genes and Genomes
LNB: Lyme neuroborreliosis
MF: molecular functions
NHP: non-human primate
PBS: phosphate-buffered saline
PD: Parkinson’s disease
PNS: peripheral nervous system
PPI: protein–protein interaction
RPKM: reads per kilobase per million reads
