# Supplementary material for: Rhesus Brain Transcriptomic Landscape in an ex vivo Model of the Interaction of Live Borrelia Burgdorferi With Frontal Cortex Tissue Explants
Source: Front Neurosci. 2019 Jun 28;13:651. doi: 10.3389/fnins.2019.00651 (PMC6610209; doi:10.3389/fnins.2019.00651)
Supplement: Supplementary file 1 [file Data_Sheet_1.ZIP › Legends.docx]

**Legends**

Additional file 1: Table S1. Profiles of differentially expressed genes (DEGs) 6 h after *Bb* infection. (XLS 1627 kb) The reads per kilobase per million reads (RPKM) of the DEGs-6h, *Bb* vs Control.

Additional file 2: Table S2. Profiles of differentially expressed genes (DEGs) 12 h after *Bb* infection. (XLS 831 kb) The reads per kilobase per million reads (RPKM) of the DEGs-12h, *Bb* vs Control.

Additional file 3: Table S3. Profiles of differentially expressed genes (DEGs) 24 h after *Bb* infection. (XLS 417 kb) The reads per kilobase per million reads (RPKM) of the DEGs-24h, *Bb* vs Control.

Additional file 4: Table S4. Gene Ontology results of the upregulated differentially expressed genes (DEGs) 6 h after *Bb* infection. (XLS 57 kb) The detailed terms enriched by ontology analysis of the upregulated DEGs-6h, *Bb* vs Control, *p* < 0.05.

Additional file 5: Table S5. Gene Ontology results of the downregulated differentially expressed genes (DEGs) 6 h after *Bb* infection. (XLS 50 kb) The detailed terms enriched by ontology analysis of the downregulated DEGs-6h, *Bb* vs Control, *p* < 0.05.

Additional file 6: Table S6. Gene Ontology results of the upregulated differentially expressed genes (DEGs) 12 h after *Bb* infection. (XLS 29 kb) The detailed terms enriched by ontology analysis of the upregulated DEGs-12h, *Bb* vs Control, *p* < 0.05.

Additional file 7: Table S7. Gene Ontology results of the downregulated differentially expressed genes (DEGs) 12 h after *Bb* infection. (XLS 41 kb) The detailed terms enriched by ontology analysis of the downregulated DEGs-12h, *Bb* vs Control, *p* < 0.05.

Additional file 8: Table S8. Gene Ontology results of the upregulated differentially expressed genes (DEGs) 24 h after *Bb* infection. (XLS 26 kb) The detailed terms enriched by ontology analysis of the upregulated DEGs-24h, *Bb* vs Control, *p* < 0.05.

Additional file 9: Table S9. Gene Ontology results of the downregulated differentially expressed genes (DEGs) 24 h after *Bb* infection. (XLS 29 kb) The detailed terms enriched by ontology analysis of the downregulated DEGs-24h, *Bb* vs Control, *p* < 0.05.

Additional file 10: Table S10. KEGG pathway enrichment analysis of FOLR2 with differentially expressed genes (DEGs) after *Bb* infection. (XLS 36 kb) The pathway enriched by PPI analysis of FOLR2 with the DEGs closely related to immunity, *p*<0.01.
